# Genome-wide characterization of human L1 antisense promoter-driven transcripts

**DOI:** 10.1186/s12864-016-2800-5

**Published:** 2016-06-14

**Authors:** Steven W. Criscione, Nicholas Theodosakis, Goran Micevic, Toby C. Cornish, Kathleen H. Burns, Nicola Neretti, Nemanja Rodić

**Affiliations:** Department of Molecular Biology, Cell Biology, and Biochemistry, Center for Computational Molecular Biology, Brown University, Providence, RI 02912 USA; Department of Pathology, Yale University, New Haven, CT 06510 USA; Department of Dermatology, Division of Dermatopathology, Yale University, New Haven, CT 06510 USA; Department of Pathology, Johns Hopkins University School of Medicine, Baltimore, MD USA; McKusick-Nathans Institute of Genetic Medicine, Johns Hopkins University School of Medicine, Baltimore, MD USA; Department of Molecular Biology and Genetics, Johns Hopkins University School of Medicine, Baltimore, MD USA; High Throughput (HiT) Biology Center, Johns Hopkins University School of Medicine, Baltimore, MD USA

**Keywords:** LINE-1, L1, Retrotransposon, Antisense promoter, EST, YY1, Transposon, Chimeric transcript, PacBio

## Abstract

**Background:**

Long INterspersed Element-1 (LINE-1 or L1) is the only autonomously active, transposable element in the human genome. L1 sequences comprise approximately 17 % of the human genome, but only the evolutionarily recent, human-specific subfamily is retrotransposition competent. The L1 promoter has a bidirectional orientation containing a sense promoter that drives the transcription of two proteins required for retrotransposition and an antisense promoter. The L1 antisense promoter can drive transcription of chimeric transcripts: 5’ L1 antisense sequences spliced to the exons of neighboring genes.

**Results:**

The impact of L1 antisense promoter activity on cellular transcriptomes is poorly understood. To investigate this, we analyzed GenBank ESTs for messenger RNAs that initiate in the L1 antisense promoter. We identified 988 putative L1 antisense chimeric transcripts, 911 of which have not been previously reported. These appear to be alternative genic transcripts, sense-oriented with respect to gene and initiating near, but typically downstream of, the gene transcriptional start site. In multiple cell lines, L1 antisense promoters display enrichment for YY1 transcription factor and histone modifications associated with active promoters. Global run-on sequencing data support the activity of the L1 antisense promoter. We independently detected 124 L1 antisense chimeric transcripts using long read Pacific Biosciences RNA-seq data. Furthermore, we validated four chimeric transcripts by quantitative RT-PCR and Sanger sequencing and demonstrated that they are readily detectable in many normal human tissues.

**Conclusions:**

We present a comprehensive characterization of human L1 antisense promoter-driven transcripts and provide substantial evidence that they are transcribed in a variety of human cell-types. Our findings reveal a new wide-reaching aspect of L1 biology by identifying antisense transcripts affecting as many as 4 % of all human genes.

**Electronic supplementary material:**

The online version of this article (doi:10.1186/s12864-016-2800-5) contains supplementary material, which is available to authorized users.

## Background

Our genome is replete with L1 retrotransposon-derived sequences that can affect the transcriptome [1–3]. Genomic L1 sequences propagate through RNA intermediates. Their lifecycle begins with transcription from the 5’ L1 promoter; this is followed by reverse transcription of the L1 RNA and insertion of the L1 cDNA sequence into the genome [1, 2]. There are two broad classes of L1s. First is the less numerous class of full-length (~6 kb) L1 retrotransposon sequences with intact internal promoters, of which there are approximately 7,000 copies in the human genome [4]. A subset of these with intact coding sequences for open reading frame 1 protein (ORF1p) and ORF2p is potentially retrotransposition competent, and these elements are mostly specific to our species (L1HS, or L1PA1 elements) [5]. Second is the much more numerous and heterogeneous class of mutated or truncated L1, which includes both species-specific and ancient insertions. These insertions included 5’ truncations with or without internal rearrangements at the time of their insertion. Depending on their length and mutation load these sequences may have no promoter activity or protein-coding capability [1, 2, 6].

It is estimated that there are approximately 90 full-length, retrotransposition competent L1HS in the human genome with intact internal promoters and open-reading frames [5]. Additionally, 29 of the 362 full-length older L1PA2 are potentially active [6]. The estimates are based on the length of the L1 insertions, the integrity of their open reading frame sequences, and results of in vitro retrotransposition assays that may use an ectopic promoter to drive L1 expression. However, sequence requirements for L1 promoter activity have not been well defined.

Activity of the L1 antisense promoter (ASP) was first demonstrated by Speek who described the discovery of four chimeric L1 ASP transcripts, comprised of L1-derived 5’-UTR and spliced exons from neighboring single-copy genes [7]. Speek and colleagues also showed that L1 ASP activity can drive tissue-specific transcription of chimeric transcripts in a few instances [8, 9]. Subsequently, much work focused on the MET oncogene locus and the associated L1-MET chimeric transcript [10]. The L1-MET chimeric transcript initiates within the second intron of the MET gene and downstream of the translational start site. Overexpression of the L1-MET chimeric transcript causes decreased full-length MET protein levels and MET-dependent signaling perhaps through transcriptional interference [11]. Similarly, transcriptional derepression of the L1 chimeric transcript LCT13 was linked to silencing of its cognate transcript TFPI-2, a tumor suppressor in a variety of human malignancies [12].

In another approach to identify chimeric L1 ASP transcripts, Cruickshanks and Tufarelli applied L1 chimera display to identify eighteen novel chimeric L1 ASP transcripts, some of which were selectively detected in breast and colon cancer specimens but not in matched normal tissues [13]. These investigators also showed that DNA methylation limits the activity of L1 ASP in normal tissues and that 5-aza-cytidine treatment of established cancer cell lines causes expression of chimeric L1 ASP transcripts [13]. Other studies have found evidence of antisense expression in more ancient L1 elements. Macia et al. found that more ancient primate L1 elements including L1PA2-10 were capable of antisense transcription [14]. Faulkner et al. identified transcription start sites of cap-selected RNAs and found that L1 fragments displayed antisense expression at the 3’ end of the L1 element [15].

The human-specific subfamily L1HS and the primate specific subfamilies L1PA2-8 were identified to contain an open reading frame termed ORF0 that is transcribed downstream of the L1 ASP on the antisense strand [16]. The ORF0 is typically translated as a short peptide and locates to promyelocytic leukemia-adjacent nuclear bodies in the cytoplasm [16]. There are ~3200 genic loci encoding L1 ORF0, which is typically encoded completely within the L1 ASP sequence. A rare fraction, 57 of ~3200 L1 ORF0 loci, encodes a peptide where ORF0 was observed to be fused to a gene exon [16].

Although the existence of L1 ASP transcripts is well-documented, many questions remain. The extent to which L1 chimeric antisense transcripts impact the human transcriptome and what other genes may be under the influence of L1 ASP is unknown. The importance of these L1 ASP transcripts is further highlighted by the recent identification of ORF0, which suggests chimeric L1 gene fusion transcripts might be expressed. Herein, we report the identification and comprehensive characterization of 988 putative L1 ASP transcripts.

## Results

### Identification of many novel L1 ASP transcripts

**Fig. 1:**
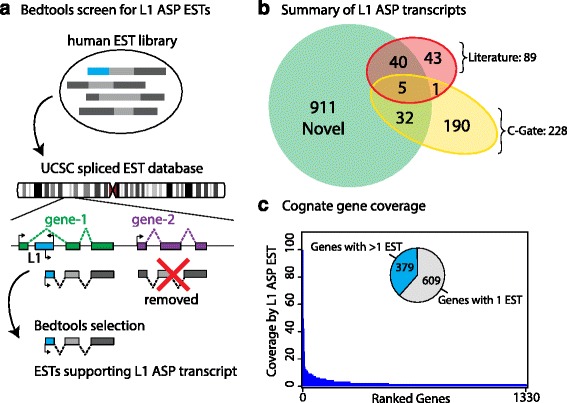
Identification of novel L1 antisense promoter (ASP) transcripts using a computational pipeline. **a** Schematic representation of our method to identify L1 ASP transcripts. The coordinates of human spliced ESTs were intersected with L1s and gene exons to identify ESTs with a TSS antisense to an L1 and overlapping an exon of a cognate gene. L1 ASP transcripts that displayed an independent EST supporting L1 exonization were removed. **b** Total number of L1 ASP transcripts identified in the current report (*n* = 988) cross-referenced to known existing L1 ASP transcripts found in published reports including recently identified L1 ORF0 gene fusions and the C-GATE database. **c** Histogram of the total number of ESTs identified per gene for L1 ASP transcripts (*n* = 2015 ESTs, some overlapping multiple genes)

We queried human expressed sequence tag (EST) genome alignments to identify ESTs incorporating part of an L1 sequence in the ASP. First, we required that the transcription start site (TSS) of a spliced human EST begins in the antisense orientation relative to an annotated L1 element and second, that a spliced exon of the EST must also overlap an annotated exon of a gene. Third, we removed putative L1 ASP transcripts where an independent EST supported L1 exonization, rather than transcription starting from within the L1 element. We implemented the third criterion because ESTs are not full-length transcripts and gene transcripts can retain intronic L1s on the opposite strand because L1s contain cryptic splice sites [17, 18]. We used BEDtools to conduct intersections and identified 2015 ESTs supporting putative L1 ASP transcripts (Fig. 1a, Additional file 1: Table S1). Only a small fraction of ESTs, 2.3 % or 46/2015, was annotated by the UCSC-spliced human EST database to map to multiple locations in the genome, with at least one these locations not matching our criteria. The 2015 ESTs map to exon sequences of 988 genes (Additional file 1: Table S1, Fig. 1b). We identified 45/89 previously known published L1 ASP transcripts [7–9, 11–13, 16] and confirmed existence of 37/228 deposited on C-GATE [19], a publicly available project on genes affected by transposable elements (Fig. 1b). The vast majority, 911/988, of L1 ASP transcripts in our survey are novel. The ESTs supporting L1 ASP transcripts were identified on every human chromosome, although the Y chromosome that is gene poor displayed relatively few chimeric transcripts (Additional file 2: Figure S1).

### Properties of L1 ASP transcripts

**Fig. 2:**
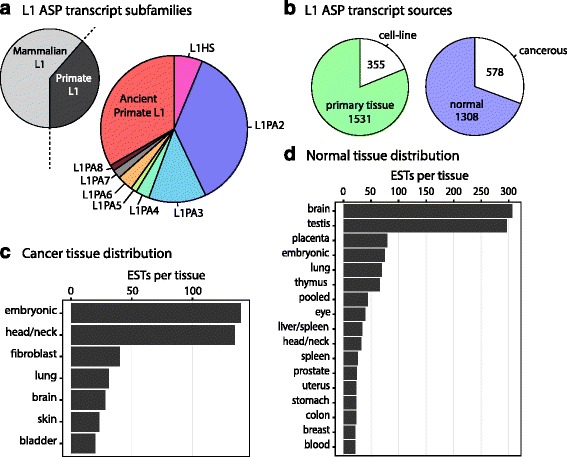
L1 subfamily and tissue distributions of L1 ASP transcripts. **a** Evolutionary age of L1 subfamilies indicates a role for most L1 evolutionary subtypes in genesis of L1 ASP transcripts. **b** Characterization of source material used for ESTs cDNA that support L1 ASP transcripts. **c** Tissue source for ESTs supporting L1 ASP transcripts identified in hyper-proliferative or cancerous samples. **d** Normal tissue sources for ESTs used to identify L1 ASP transcripts

Of the L1 ASP transcripts, 609 were represented by only one EST, which suggests that many L1 ASP transcripts are transcribed at relatively low levels. The remaining 379 transcripts were represented by multiple ESTs (Fig. 1c). The top three most widely expressed L1 ASP transcripts were associated with CPM (99 ESTs), BCAS3 (49 ESTs), and DDX39B (42 ESTs). Interestingly, L1-CPM has not been previously identified. We next annotated the identified L1 ASP transcripts by the subfamily of L1 that contained the ASP. Remarkably, we found that, of the 2015 identified ESTs, only 52/2015, or 2.6 % of all of the transcripts, originate from the evolutionarily young L1HS subfamily (Fig. 2a, Additional file 2: Figure S2). The vast majority, 626/2015 or 31.1 %, originate from pre-Ta, primate-specific L1 subfamilies and 1337/2015 or 66.3 % originate from older mammalian L1 subfamilies. The prevalence of evolutionarily ancient and mutated L1s as a source of L1 ASP transcripts was unexpected and suggests the possible exaptation of the L1 sequences over evolutionary time.

Next, we reviewed the primary tissues (1531 ESTs) and cell lines (355 ESTs) that were sources of ESTs, and categorized them as cancerous (578 ESTs) or normal (1308 ESTs) (Table S1). Some ESTs could not be characterized due to a lack of supporting information. Interestingly, 69.4 % of ESTs supporting an L1 ASP transcript originated from non-cancerous tissues (Fig. 2b). For ESTs derived from cancerous tissue, the most prevalent sources were embryonic carcinoma cells (NT2 testis embryonal carcinoma cell line), head/neck tissue (tongue tumor), and fibrosarcoma cells (HT1080 fibrosarcoma cell-line) (Fig. 2c). L1 ASP transcripts in normal tissue were primarily identified from brain or testis tissues. Our analysis supports the notion that L1 ASP transcripts are readily detectable in both normal and diseased states. However, due to source tissue biases in the EST database, we cannot conclude the L1 ASP promoter activity is higher overall in the brain or testis. Finally, we conducted a gene ontology (GO) analysis of the 988 cognate genes that contained putative L1 ASP transcripts. The results of our GO analysis showed overrepresentation of genes involved in diverse cellular processes including vesicle-mediated transport, intracellular protein transport, mitosis, morphogenesis, and protein modifications (Additional file 3: Table S2).

**Fig. 3:**
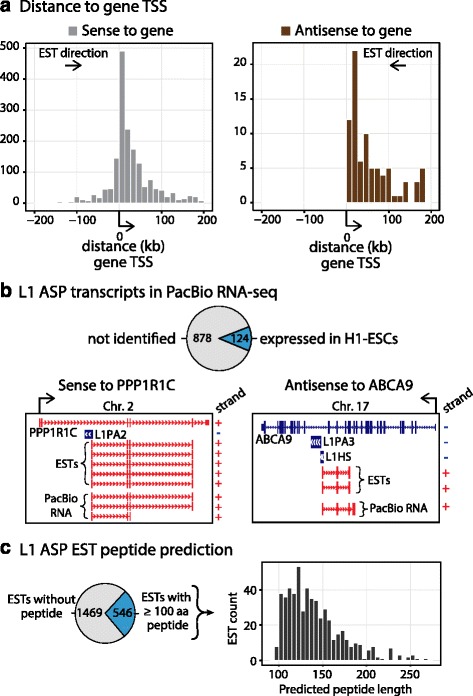
Most L1 ASP transcripts are sense to cognate genes and possess protein coding potential. **a** L1 ASP transcripts tend to be in sense strandedness relative to the cognate gene, typically serving as an alternative TSS of the canonical gene TSS (left panel). Rarely, ESTs supporting L1 ASP transcripts overlap anti-sense to cognate gene (right panel). The bias towards sense-strandedness of L1 ASP transcripts suggested the possibility for protein coding potential. ESTs supporting L1 ASP transcripts that had multiple alignments in the database (~2.3 % of all ESTs) were excluded. **b** Independent analysis of long-read PacBio RNA-seq data identified transcript isoforms supporting 124/988 L1 ASP transcripts. We validated both instances where the L1 ASP transcript was sense to the cognate gene (L1-PPP1R1C, left panel). We also validated the opposite strand L1 ASP transcript (L1-ABCA9, right panel). The red indicates the positive strand and the blue indicates the negative strand for the genome browser view. **c** Coding potential of L1 ASP transcripts was assessed by the ability to encode an open reading frame (ORF) of at least 100 amino acids (aa) and to begin with a start codon. We identified that 27.1 % of ESTs contained the potential for coding by these criteria

We next examined the relationship of ESTs supporting L1 ASP transcripts to their overlapping cognate genes. Some of the 2015 ESTs overlapped multiple genes; in total, we identified 2316 pairwise overlaps between the ESTs and an annotated gene exon. We found that the vast majority of chimeric transcripts, 2134/2316 or 92.1 %, were sense to the nearby gene, a proportion that was highly statistically significant (p-value, < 2.2*10^−16^, chi-squared test); on the contrary, only 182/2316 or 7.9 % were in the antisense orientation. We examined the distance from the EST transcription start site (TSS) to the TSS of the cognate gene in these two categories. The L1 ASP transcripts sense to their cognate genes was typically downstream of the gene TSS. Thus, the L1 provides an alternative start site and produces a transcript in which the annotated 5’UTR or first coding exon is not included (Fig. 3a, b left panels). All of the L1 ASP transcripts that were on the strand opposite from the cognate gene were downstream of the gene TSS (Fig. 3a, b right panels). This minor category of L1 ASP transcripts extended and overlapped antisense to the exon of the cognate gene.

To further validate L1 ASP transcripts we examined publicly available long-read RNA-seq data from human embryonic stem cells (H1-ESCs) sequenced using a Pacific Biosciences (PacBio) instrument [20]. The RNA-seq reads generated by PacBio are long (averaging 2–3 kb length), but are lower quality. Therefore, Au K. et al. error corrected the long-read PacBio RNA-seq reads using short read Illumina RNA-seq data [20]. Subsequently, the error corrected long RNA-seq data was used for isoform detection by Au K. et al. to identify expressed transcripts in H1-ESCs [20]. We used the publicly available transcript isoform prediction for H1-ESCs to validate L1 ASP transcripts identified by our EST screen. We were able to validate 124/988 or 12.6 % of the L1 ASP transcripts reported here as expressed in H1-ESCs (Fig. 3b, Additional file 1: Table S1). Thus, using low coverage RNA-seq data from a single cell-line we were able to independently validate 12.6 % of the L1 ASP transcripts we identified.

Next we sought to address the coding potential of L1 ASP transcripts because the majority was sense to the cognate gene and could potentially contain open reading frames (ORFs). We applied TransDecoder software to identify putative peptides encoded by ESTs with at least 100 amino acids (aa) [21]. We also required that the predicted putative peptide begin with a start codon encoding methionine and identified that 546/2015 or 27.1 % of the ESTs contained the potential for coding putative peptides (Fig. 3b). The identified putative peptides ranged from 100 to 268 aa, with an average putative peptide length of 140 aa (Additional file 4: Table S3, Fig. 3b). Of the identified putative peptides 42 contained fragments of the recently identified ORF0 open reading frame protein [16]. However, because we only included spliced ESTs and ORF0 is contained within the unspliced 5’ UTR of L1HS we did not identify a full-length ORF0 [16]. However, it is important to note that in the absence of additional experimental data these are only peptide predictions. In summary, L1 ASP transcripts are predicted to contain putative peptides that extend from the L1 ASP into cognate genes.

### Characterization of the L1 ASP

**Fig. 4:**
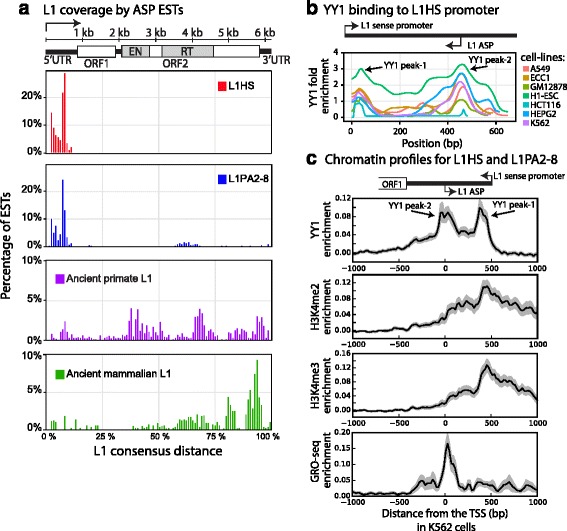
Features of the L1 antisense promoter revealed from ESTs, ENCODE ChIP-seq, and GRO-seq data. **a** ESTs supporting alternative L1 ASP transcripts for L1HS, L1PA2-8, ancient primate L1, and ancient mammalian L1 subfamilies were aligned to full-length L1 consensus sequences. The plot revealed evolutionarily recent L1HS and L1PA2-8 subfamilies possess the ASP activity in the 5’ UTR, whereas ancient primate and ancient mammalian L1 subfamilies display minor ASP activity in the 5’ UTR and the majority is at the 3’ end of the element overlapping the end of ORF2. **b** YY1 enrichment profile in the 5’UTR of L1HS consensus sequence for ENCODE YY1 ChIP-seq in various cell-lines. The schematic above is a reference point to the L1HS consensus position. **c** The TSS enrichment profile for YY1, H3K9me2, and H3K9me3 ChIP-seq for L1HS and L1PA2-8 AS ESTs in units of input subtracted reads per million mapping reads (RPM) using the K562 cell-line. The bottom panel displays the GRO-seq TSS enrichment profile in RPM enrichment units. The schematic above is a reference point to the L1HS consensus position

Next we compared the L1 ASP transcription start sites to the L1 consensus sequences. We aligned the 2015 ESTs supporting L1 ASP transcripts to full-length consensus sequences in RepBase and those reported by Khan et al. [4]. We divided the L1s by evolutionary age, examining the most recent human L1HS, less recent primate L1PA2-8, more ancient primate L1, and ancient mammalian L1 subfamilies. We observed that the ESTs mapping to L1HS elements aligned within the first 600 bp of the 5’ UTR antisense to the L1 consensus sequence (Fig. 4a). The locations of the L1HS antisense TSS within the 5’ UTR were consistent with prior descriptions of L1 ASP transcripts [7]. Recent primate L1PA2-8 elements that are highly homologous to L1HS in the 5’ UTR [22] also displayed L1 ASP activity in an identical location to L1HS (Fig. 4a). However, older primate L1s and ancient mammalian L1s displayed only minor L1 antisense activity in the 5’ UTR and the majority of activity was in the 3’ end of the element overlapping ORF2 (Fig. 4a). Our result is consistent with a prior report demonstrating that L1 fragments display ASP activity at the 3’ end of the element [15].

We further interrogated ESTs from recent primate L1PA2-8 subfamilies and the human L1HS subfamily for transcription factors that might drive transcription from the ASP [22]. YY1 is the best characterized cis-regulatory transcription factor proposed to be required for L1 transcription from the sense promoter [23]. YY1 was previously reported to bind between 13 and 21 bp of L1HS 5’ UTR on the antisense strand [23–26]. Interestingly, YY1 is also an important regulator of bidirectional transcription at the promoters of multiple single-copy genes [27, 28]. To examine the possibility that YY1 could play a role in the L1 ASP, we sought to better characterize YY1 binding to the L1HS 5’ UTR. First, we applied JASPAR Scan to examine the 5’ UTR of the L1HS consensus sequence for YY1 transcription factor-binding sites using a relative score of at least 90 % [29]. We identified five putative binding sites based on YY1 position weight matrices, including two that overlapped the proposed binding site (13 to 21 bp) and three additional binding sites (Additional file 5: Table S4). Next, we examined YY1 transcription factor binding to the L1HS consensus sequence using ChIP-seq data available for YY1 from the ENCODE project [30]. For all of the ENCODE cell lines, we identified two peaks that displayed enrichment for YY1: the first, Peak-1, corresponded to the known YY1 binding site at ~20 bp; the second, Peak-2, was at ~450 bp in the L1HS 5’ UTR (Fig. 4b). Peak-2 overlaps closely with an identified putative YY1 binding site on the positive strand at 448 to 453 bp, which immediately precedes the primary L1 ASP at ~400 bp. (Fig. 4a-b). The secondary binding site of YY1 near the primary L1 ASP suggests that YY1 may play a role in regulating L1 antisense transcription.

To test whether TSS locations of L1 ASP transcripts were bound by YY1 and marked by histone modifications, we examined ENCODE ChIP-seq alignments to the human genome (build hg19). We reviewed ChIP data for YY1, H3K4me2, and H3K4me3 histone modifications in the K562, H1-ESC, and Hela cell-lines [30]. The histone modifications H3K4me2 and H3K4me3 are typically associated with euchromatic, active or poised promoters [31–34]. We found that the ESTs supporting L1 ASP transcripts displayed modest enrichment for YY1 at the TSS in ENCODE (Fig. 4c, Additional file 2: Figure S3). Peak-2 for YY1, identified in Fig. 4b, directly overlapped near the TSS of the ESTs supporting L1 ASP transcripts. Immediately downstream of the TSS, based on transcription orientation, we identified modest enrichment for histone marks H3K4me2 and H3K4me3 in K562, H1-ESC, and Hela cell-lines (Fig. 4c, Additional file 2: Figures S4A-B). We next examined global run-on sequencing (GRO-seq) data, a high-throughput nuclear run-on assay that can identify 5’-capped RNAs [35–37]. We found that GRO-seq sequencing reads were mapped to the TSS of the majority of L1 antisense ESTs in K562, MCF7, and Hela cell-lines (Fig. 4c, Additional file 2: Figures S4C-D). Together, this data supports that YY1 binds to the L1 ASP in addition to the previously characterized binding site near the sense promoter. Additionally, analysis of histone modifications associated with active or poised promoters and GRO-seq data suggests that the L1 ASPs we identified are likely to be actively transcribed in a variety of cell types.

### Validation of L1 ASP transcripts

**Fig. 5:**
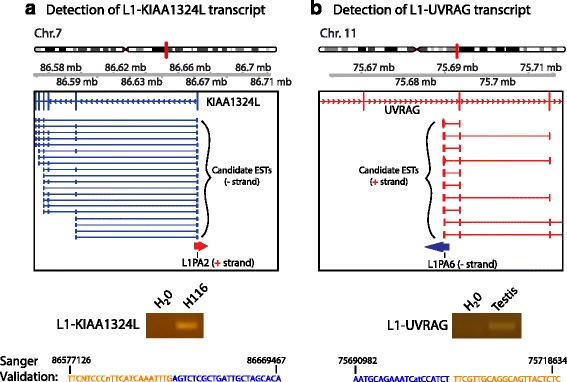
Validation of selected L1 ASP transcripts by combined PCR and Sanger-sequencing methods. **a** Upper panel: Genome browser view of KIAA1324L genomic locus displaying the ESTs supporting L1- KIAA1324L. Middle panel: Representative PCR product obtained by PCR amplification with L1-specific primer and KIAA1324L-specific primer. Lower panel: selected Sanger sequencing read across L1 to KIAA1324L exon 1 boundary; blue letters denote L1 AS promoter sequence, orange letters denote KIAA1324L exon 1 sequence. **b** Empirical validation of L1-UVRAG, with same orientation as Figure 5a

**Fig. 6:**
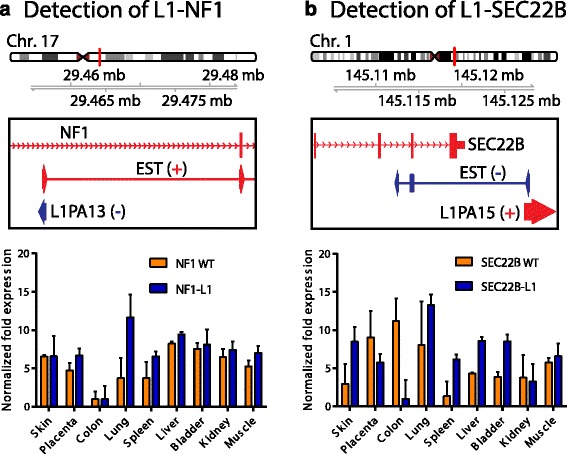
L1 driven transcripts are expressed in many normal human tissues. **a-b** Tissue specific expression levels of L1 ASP transcripts L1-NF1 and L1-SEC22B relative to wild-type cognate genes, respectively. Upper panel: Genome browser view of genomic locus displaying the ESTs supporting L1 ASP transcripts. Lower panel: Quantitative RT-PCR results of L1 ASP transcripts and cognate wild type gene in 9 tissues

We selected two genes associated with L1 ASP transcripts for Sanger sequence-based confirmation. We successfully detected L1 ASP transcripts associated with both KIAA1324L and UVRAG genes (Fig. 5a-b, Additional file 6: Table S5). Sanger sequenced reads across L1-KIAA1324L and L1-UVRAG transcripts revealed that L1 antisense was fused in frame to KIAA1324L and UVRAG. We compared our list of genes associated with L1 ASP transcripts to the genes causally implicated in human malignancies annotated by the COSMIC database [38, 39]. We identified 20 implicated causal cancer-associated genes with L1 ASP transcripts, including L1-MET, L1-JAK1, L1-NF1, L1-PRKAR1A, and L1-RHOA (Additional file 7: Table S6). Apart from L1-MET and L1-CBFA2T3, the other 18 cancer-associated L1 ASP transcripts have not previously been described. Interestingly, L1-NF1 expression in normal human tissues closely resembled the expression of cognate cancer-associated gene NF1 (Fig. 6a). The validation of the L1 antisense transcript for SEC22B also showed that the transcript was expressed at levels near wild-type cognate SEC22B transcripts (Fig. 6d). The L1-SEC22B and L1-NF1 were each supported by one EST, whereas ~118 and ~771 spliced ESTs corresponded to the wild type SEC22B and NF1 transcript, respectively. The discrepancy between EST ratios and quantitative RT-PCR results can be explained by the heterologous mixture of tissues and cDNA library size used as source material for ESTs. Many ESTs supporting the wild-type transcripts have identical alignment coordinates and are from the same cDNA library. These issues complicate the quantitation of EST ratios and make comparison of these ratios to the quantitative RT-PCR results difficult to interpret. Although we note this discrepancy, the validation of 4/4 L1 ASP transcripts by quantitative RT-PCR and Sanger sequencing supports that the screen identified many new and undescribed chimeric transcripts that are driven by the L1 ASP.

### L1 ASP transcripts overlapping L1 exonized transcripts

The method to identify L1 ASP transcripts implemented a criterion to remove ESTs where an independent EST suggested L1 exonization (e.g., inclusion of an intronic L1 on the opposite strand within a normal gene transcript). We examined this category in more detail and report these putative transcripts separately (Additional file 8: Table S7). Many of these events cannot be unambiguously assigned as an L1 antisense transcript due to the presence of an EST also supporting L1 exonization. However, we observed transcripts where the majority of ESTs supported L1 ASP driven transcription rather than L1 exonization (including MAPK10, Additional file 2: Figure S5A). We also observed events where ESTs supporting L1 ASP driven transcripts and ESTs supporting L1 exonization were non-overlapping (including SCAMP1, Additional file 2: Figure S5B). Therefore, in some cases L1s might contribute both to L1 ASP driven transcripts and L1 exonized transcripts.

### Identification of mouse L1 ASP transcripts

In the mouse genome LINE-1 elements are also active, yet they are quite different from the human L1HS subfamily at the sequence level. Nevertheless, active mouse LINE-1 elements contain an L1 ASP also capable of yielding fusion transcripts [40]. Unlike the human L1HS subfamily, which contains the L1 ASP in the 5’ UTR, the mouse ASP is within the first open reading frame (ORF1p) [40]. We applied our bioinformatic approach to identified spliced ESTs consistent with an L1 antisense transcript in the mouse genome. The results for the mouse are an underestimate because unlike human ESTs many of the mouse ESTs in the spliced alignment database did not contain transcript orientation. However, despite our strict filtering we identified L1 ASP transcripts for 174 cognate genes that were supported by 307 ESTs (Additional file 9: Table S8). Of the 174 identified L1 ASP transcripts 23 corresponded to the mouse-specific LINE-1 retrotransposon subfamilies (L1Mus1-4). Surprisingly, we again identified a subset of evolutionarily ancient L1 subfamilies that were transcribing an L1 antisense transcript, which we also identified in human. The L1 ASP transcript SHISA5 was identified within both the human and mouse genomes transcribed antisense to shared mammalian L1M5 (Additional file 2: Figure S6).

## Discussion

Here, we present one of the most comprehensive studies of L1 ASP transcripts in the human genome. We describe in total 988 chimeric transcripts of which 911 are novel, where the L1 ASP drives the expression of a transcript that is spliced to a gene exon; and they are collectively supported by 2015 ESTs. Because we required evidence of splicing at the alignment level, our identified L1 antisense ESTs correspond to processed transcripts. Thus, the number of putative transcripts is almost certainly an underestimate. This is supported by the fact that other published studies using complementary approaches identified 44 additional putative L1 ASP transcripts we did not recover.

The L1 sense promoter has higher transcriptional activity in many cancers, and increased L1 ORF1 expression and protein abundance is often observed [41, 42]. High levels of L1 somatic retrotransposition are also readily detectable in a variety of cancers and during the early stages of tumorigenesis [43–49]. We identified 20 L1 ASP driven transcripts that affect cancer genes, including previously reported L1-MET [11]. While we expected to identify an increased number of L1 ASP transcripts from cancer specimens, the majority of ESTs found in this survey were identified in normal tissues. This is unlikely due to a bias in the EST database, which contains a preponderance of cDNAs from cancerous tissue and diseased states. Our analysis revealed that the brain, testis, placenta, embryonic tissues, and lungs were the most abundant contributors of ESTs supporting L1 antisense transcription.

About 27 % of the L1 ASP transcripts we describe occur in the sense orientation to the cognate gene and are predicted to produce a peptide of at least 100 amino acids in length. Whether L1 ASP transcripts yield translated proteins remains an open question. However, recent characterization of ORF0 and 57 instances of ORF0 gene exon fusions supports that a subset of transcripts identified here are likely translated [16]. Interestingly, some L1 ASP transcripts seem to match an annotated gene transcript; in those cases the gene TSS starts in the L1 ASP, an example being UVRAG.

In contrast, about 73 % of the L1 ASP transcripts are not predicted to encode a putative peptide by our metric. The absence of a predicted putative peptide of 100 amino acids has been previously used to define a transcript as a long non-coding (lnc) RNA [50–52]. However, although atypical, some proteins with less than 100 amino acids reside in mammalian genomes [53]. An important example of a short peptide that would not be identified by this analysis is ORF0, which is only 71 amino acids [16]. Hence, our putative peptide prediction does not preclude the possibility that additional L1 ASP transcripts are potentially protein coding. In addition, because our identification is based on ESTs, which are not typically full-length transcripts, our current list might represent an underestimate of L1 ASP transcript putative predicted peptides.

We also describe L1 ASP transcripts incorporating gene sequences in the antisense orientation. Such transcripts are rare, comprising less than 8 % of our total, but may be biologically important. Where expressed, these have the potential to produce double-stranded RNAs because they encode a transcript with reverse complementarity to a portion of the cognate mRNA (for example, RABL2B in Additional file 1: Table S1). The dsRNA may impact epigenetic regulation at the locus and the stability of the mRNA [54].

Nearly all of the L1HS and L1PA2-8 antisense ESTs, subfamilies with a homologous 5’ UTR, display ASP activity within the first 600 bp of the L1 5’ UTR, whereas more ancient primate and mammalian L1s display the majority of ASP activity at the 3’ end of the element near the end of ORF2. This observation explains a previous result indicating that L1 fragments display ASP activity at the 3’ end of the L1 element [15]. In addition, this result clarifies how the majority of ancient L1s, which are typically 5’ truncated, can contribute the majority of L1 ASP activity. We identified substantial evidence that L1 ASPs are transcriptionally active. There was binding of regulatory elements including the transcription factor YY1 at the L1 ASP. The transcription factor YY1 displayed a double peak binding distribution within the L1 promoter. YY1 transcription factor peak-1 in Fig. 4-c corresponds to the position of the L1 sense promoter and has been described previously [23]. YY1 transcription factor peak-2 is newly identified and seems to overlap the position of the L1 ASP; however, binding of YY1 to peak-2 does not necessarily indicate a functional role. Unlike YY1 the GRO-seq and histone mark ChIP-seq profiles display a single peak distribution within the L1 promoter (Fig. 4c). The single GRO-seq peak overlaps YY1 peak-2 at the site which is likely the TSS for the L1 ASP. The predicted TSS position is also marked downstream by histone modifications H3K4me2 and H3K4me3 which is characteristic of active promoters. The GRO-seq profiles are consistent with 5’ capped RNAs initiating from this L1 ASP TSS. Whether the YY1 peak-2 is required for L1 ASP transcription at the identified TSS warrants further investigation. Independent validation of 124/988 L1 ASP transcripts using PacBio long read RNA-seq suggested expression of a large subset of identified transcripts in embryonic stem cells. Together, several lines of evidence indicate these transcription start sites function as active promoters.

The L1 ASP was likely active at multiple points in mammalian evolution. While ancient subfamilies are no longer competent for retrotransposition they are contributing to the transcriptome through promoter activity. Similarly, in the mouse, L1_Mus1-4 subfamilies also contribute to new L1 ASP transcripts. The mouse element contains the ASP in ORF1p [40]. While the sequence differs dramatically between human and mouse L1s, the functional conservation of ASP activity indicates selective pressure to preserve this feature. It is interesting to speculate as to whether the L1 ASP activity benefits the host or the L1 element fitness. There is some evidence that the L1 ASP transcripts might produce siRNAs to repress the L1 sense transcript [55]. Contradicting this view is new evidence that ORF0 transcribed from the ASP is correlated with the transposition competent sense transcript [16]. Lastly, there are more instances being identified of transposons being exapted for normal cellular functions by the host [56]. The fact that some ancient mammalian L1 elements are conserved in diverse mammals from human to mouse provides support to the hypothesis that L1 ASP transcripts may be exapted for functional roles.

Perhaps one of the most interesting and easily testable ramifications stemming from identification of such a large number of L1 ASP transcripts is that some are likely to be polymorphic in the human population. Some of the transcripts identified here are likely to be absent in at least some individuals in the human population. By extension, there are likely polymorphic L1 transposition events present in the population but absent from the reference genome that would also likely contribute to L1 ASP transcripts. It would be of interest to determine allele frequency for a handful of chosen L1 ASP transcripts. For instance, is the putative L1-MET chimeric transcript polymorphic, and does it segregate with any observable phenotype? The expanded repertoire of L1 ASP transcripts described herein could exert numerous effects on gene regulation that remain to be investigated.

## Methods

### Computational detection of putative L1 ASP transcripts

We identified candidate L1 ASP transcripts by applying a series of intersections on genomic intervals of three annotations obtained using the UCSC table browser tool in Browser Extensible Data (BED) format. First, we downloaded the locations of L1 elements by extracting the coordinates of all LINE-1 family repeats from the Repeatmasker (hg19) annotation [57]. Second, we downloaded the annotated exon coordinates of RefSeq genes (hg19). Third, we downloaded the coordinates of UCSC human spliced ESTs, a table containing the alignment coordinates of ESTs displaying evidence of splicing, as a block feature (start to end) and as coordinates of spliced EST exons (downloaded July 2015). Importantly, the strand field in the UCSC human spliced ESTs table reflects the alignment direction (e.g., plus is 5’ to 3’) of the EST cDNA to the genome by Blat and does not reflect the transcriptional direction. To convert the strand in the UCSC human spliced ESTs database to reflect transcriptional direction, we used the linked UCSC estOrientInfo table. If the value of the intronOrientation field in the associated table is positive, the transcriptional direction matches the alignment direction; however, if the intronOrientation field is negative, the strandedness is opposite. Sometimes, a call cannot be determined for EST transcriptional direction because the intronOrientation field is zero; these ambiguous cases were removed manually from subsequent analyses. For mouse we used a similar set of annotations: Repeatmasker (mm10), RefSeq genes (mm10), and the UCSC mouse spliced ESTs (mm10, downloaded July 2015). For the mouse annotation, a larger fraction of cases were ambiguous and removed because the intronOrientation field was zero and transcriptional direction could not be determined.

Next, we defined criteria to identify putative L1 ASP transcripts. The 5’ TSS of the EST was required to originate from within an annotated L1 element, and the transcriptional direction strand of the EST was required to be antisense to the L1. Second, an exon coordinate of the EST was required to overlap with an annotated exon coordinate of a gene. Third, we excluded L1 ASP transcripts where an independent EST supported L1 exonization (e.g., inclusion of an intronic antisense L1 in a gene transcript of the structure exon to L1 to exon). We selected ESTs that met this criteria and represented L1 ASP transcripts using sequential intersections performed with bedtools [58]. To verify the accuracy of the selection method, we manually inspected putative L1 ASP transcripts using the UCSC genome browser. Manual inspection identified a subset of ESTs that contained a higher degree of uncertainty due to the presence of multiple annotated alignments. We subsequently added whether the UCSC human spliced ESTs contained more than one alignment of the EST to Additional file 1: Table S1, in order to distinguish this category.

### Computational validation with PacBio RNA-seq

The long-read PacBio RNA-seq data from human embryonic stem cells (H1-ESCs) was annotated as transcript isoform predictions by Au, K. et al. [20]. We downloaded H1-ESCs transcript isoform predictions in GTF format and converted the data to BED format. We applied the same intersections as described above for the EST screen using BEDtools. We characterized the filtered set of PacBio transcript isoforms for those also identified by the EST screen that supported L1 ASP transcripts.

### Annotation of ESTs that support L1 ASP transcripts

Additional information on tissue of origin, cell line of origin, and normal/cancer status were obtained by extracting and parsing associated information on ESTs downloaded from the Batch Entrez portal (http://www.ncbi.nlm.nih.gov/sites/batchentrez), which was then added to Additional file 1: Table S1. The ESTs supporting putative L1 ASP transcripts may be represented multiple times in Additional file 1: Table S1 because they overlap with more than one gene; however, further data analysis was only conducted on the unique set of ESTs identified. We manually inspected 554 of the identified ESTs using the UCSC genome browser and verified they all matched the above criteria for an L1 ASP transcript in at least one location. The EST sequences of all identified putative L1 ASP transcripts were also downloaded in FASTA format using the Batch Entrez portal. The sequences were examined for the presence of open reading frames (ORFs) of at least 100 bp using the TransDecoder module (http://transdecoder.github.io/) of Trinotate Transcript Annotation, which is a part of the Trinity package [21]. We also required that predicted ORFs start with a start codon encoding methionine to be considered a putative peptide. The genes associated with putative L1 ASP transcripts were used for gene ontology analysis using the PANTHER online statistical overrepresentation test [59] and the PANTHER GO slim biological process (with redundant GO categories removed). The raw p-values for the full results reported by PANTHER were corrected using Benjamini Hochberg false discovery rate correction using the R statistical language [60].

### Characterization of the L1 ASP

We examined L1 ASP transcripts in four categories: human-specific L1HS, primate-specific L1PA2-8, ancient primate L1s, and ancient mammalian L1s. The EST sequences were aligned to full-length consensus sequences of L1s in RepBase and those reported by Khan et al. [4] using the LAST aligner (http://last.cbrc.jp/) [61]. For each EST the position of the TSS was computed as a percentage alignment position with respect to the full-length consensus. YY1 transcription factor-binding sites in the 5’ UTR were identified by extracting the 5’ UTR sequence from the L1HS consensus FASTA. The online tool JASPAR Scan was used to identify YY1 binding sites corresponding to two PWM for YY1 (MA0095.1 and MA0095.2) against the first one kb 5’ UTR of the L1HS consensus using a relative score threshold of 90 % [29]. To create YY1 transcription factor profiles, cell lines in the ENCODE project, for which YY1 ChIP-seq was performed, were selected (for the full list of publically available data, see Additional file 10: Table S9). The YY1 ChIP-seq and input control reads were aligned to the L1HS consensus sequence using bowtie1, which is ideal for short reads <100 bp [62]. The log2FC enrichment of YY1 was calculated per-base-pair of L1HS consensus using the read coverage per million mapping reads (RPM) of YY1 ChIP and input control, for which the normalizing factor, total number of mapping reads in the library, was determined by separate alignment to the human genome (build hg19). The raw log2FC YY1 enrichment per-base-pair signal of L1HS consensus was smoothed by applying LOESS smoothing with parameter α = 0.1.

To build TSS profiles of L1 ASP transcripts, we obtained the TSS coordinate for L1PA2-8 and L1HS antisense EST transcripts for plus-strand or minus-strand ESTs. We downloaded alignment files from the ENCODE data repositories in BAM format (genome build hg19, see Additional file 9: Table S8 for a complete list) for the K562, H1-ESC, and Hela cell-lines and ChIP-seq data for the H3K4me2 and H3K4me3 histone marks and associated input controls. For K562 cells, we also downloaded alignments for YY1 ChIP-seq data and input controls. The average ChIP enrichment of EST TSS for plus- and minus-strand L1 ASP transcripts was calculated using Python package Metaseq using a genomic window of +/−1000 bp TSS and a 100 bp bin size to calculate depth [63]. The results for the plus and minus strands were merged, where −1000 bp represented upstream of the TSS and +1000 bp downstream of the TSS. The output of Metaseq was the input-subtracted ChIP enrichment normalized as RPMs. The GRO-seq data were analyzed in a similar manner (see Additional file 10: Table S9 for a complete list); however, alignments to the human genome (build hg19) were conducted using BWA-MEM [64]. We used Samtools to separate the positive-strand and minus-strand GRO-seq reads and analyzed these reads against plus strand or minus strand EST TSSs using Metaseq [65]. The results for the plus- and minus-strand GRO-seq profiles were merged to obtain normalized RPM enrichment.

### Tissue specimens

For each specimen, we procured a single tissue fragment, measuring ~ 1.0 × 0.5 × 0.5 cm, of selected grossly unremarkable organs. All tissues were stored at −80°C.

### Validation of selected chimeric L1 ASP transcripts

We selected L1-UVRAG and L1-KIAA1324L chimeric L1 ASP transcripts for validation. Using semi-quantitative RT-PCR, we amplified a region across the putative chimeric L1 ASP transcript that spans the L1 promoter and nearby exon. Half of the reaction (10 μl) was resolved on a 2 % agarose gel to confirm the presence of a singular PCR product. The remainder of the reaction was cloned using a TOPO TA Cloning Kit according to the manufacturer’s specifications (Thermo Fisher Scientific, Inc.; Wilmington, DE) and sequenced via Sanger sequencing.

### RNA isolation and cDNA synthesis

We originally yielded very small RNA amounts (<20 ng RNA total) from fibrous organs, such as skin and muscle. Therefore, we developed an in-house RNA isolation method by modifying the protocol of an RNAeasy Plus Mini Kit (Qiagen Sciences, Inc.; Germantown, MD). First, a razor and forceps were used to finely mince one small tissue fragment, weighing up to 50 mg, on dry ice. The minced tissue fragments were suspended in 900 ml of PBS, to which 100 μl of collagenase/dispase solution (stock at 10 mg/ml) was added. The solution was incubated at 37 °C for 1 h. Then, the lysate was centrifuged for three (3) minutes at full speed using a bench-top centrifuge. The pellet was collected and mixed with 10 μl of β-mercaptoethanol and 1 ml of TRIzol reagent. The specimen on ice was homogenized using a hand-held homogenizer for 5 takes, each entailing continuous homogenization for 1 min followed by rest for 30 s. The specimen was incubated for 5 min at room temperature. Proteinase-K was then added to a final concentration of 250 μg/ml (which came out to 12 μl of 20 mg/ml stock) and incubated at 56 °C for 10 min [66]. The lysate was pipetted directly into a QIAshredder spin column and centrifuged for 2 min at full speed. The supernatant was collected into a gDNA Eliminator spin column; the specimen was centrifuged for 30 s at 8,000 x g, and the flow-through was saved. The RNA precipitation was initiated by adding 0.25 ml of chloroform to the supernatant, and the specimen was shaken vigorously for 30 s. The specimen was incubated at room temperature for 10 min, followed by centrifugation for 5 min at 12,000 x g at 4 °C. The aqueous layer was separated, without touching the middle layer (interface), and then mixed with 2 μl of Pellet Paint NF Co-Precipitant reagent. 1 volume (usually ~200 μl) was mixed with ice-cold 70 % isopropanol into the specimen, and precipitate was allowed to form for 10 min at room temperature. The RNA was pelleted by centrifugation at maximum speed for 15 min, after which the supernatant was removed carefully. The pellet was in 500 μl of 70 % ethanol, to remove as much of the overlying supernatant as possible, and then air dried at room temperature for approximately 5 min. Finally, the pellet was suspended in 30–50 μl of RNASE-free deionized water and the RNA was quantified via a NanoDrop 1000 Spectrophotometer (Thermo Fisher Scientific, Inc.; Wilmington, DE). cDNA were synthesized using poly-T primers according to the manufacturer’s specifications (Roche Diagnostics; Basel, Switzerland).

### Quantitative real-time PCR

We estimated the relative abundance of targeted RNAs by resulting traditional Cq (threshold cycle; quantitative cycle) interval values in a StepOnePlus™ Real-Time PCR System (Thermo Fisher Scientific, Inc.; Wilmington, DE). Each experiment was performed in technical triplicates. Actual PCR products were quantified with a FastStart Universal SYBR Green probe (Roche Diagnostics; Basel, Switzerland). The relative abundance of experimental RNA—specifically, the arithmetic means of the Cq values—was normalized to that of an internal control RNA (GAPDH) to relative PCR efficiencies and pictorially represented as Delta Cq (ΔCq) means ± standard deviation. The PCR primers used in this report are in Additional file 11: Table S10.

## Conclusions

We identify 988 putative L1 antisense chimeric transcripts, the vast majority of which are novel. We independently verify some L1 antisense chimeric transcripts using both bioinformatics analysis and empirical evidence. Interestingly, some L1 antisense chimeric transcripts are associated with evolutionarily ancient L1 subfamilies, suggesting exaptation of these evolutionarily-older L1 sequences. We conclude that L1 antisense promoters contribute to the transcription of up to 4% of all human genes and may potentially have wide-ranging effects in health and disease.

## Abbreviations

AS, Antisense; ASP, Antisense promoter; CAGE-seq, Capped analysis of gene expression sequencing; ChIP, Chromatin immunoprecipitation; EST, Expressed sequence tag; GO, Gene ontology; GRO-seq, Global run-on sequencing; L1, Long Interspersed Element-1; LINE-1, Long Interspersed Element-1; lncRNA, Long noncoding RNA; ORF, Open reading frame; TSS, Transcription start site; UTR, Untranslated region

## Additional files

Additional file 1: Table S1. (excel) Summary of genomic screen result identifying ESTs supporting putative L1 antisense promoter (ASP) transcripts in the human genome (hg19). The 2015 ESTs are sometimes represented more than once in Table S1 because a single EST can overlap multiple cognate genes. (XLSX 276 kb) Additional file 2: Supplementary figures 1–6 (PDF). Figure S1. Genomic coordinates of 2015 ESTs supporting L1 ASP transcripts. A) ESTs supporting L1 ASP transcripts from an evolutionarily ancient primate or mammalian L1. B) ESTs supporting L1 ASP transcripts from an evolutionarily recent human-specific L1HS or primate-specific L1PA2-8 subfamily. Figure S2: Evolutionary age of L1 subfamilies that contribute to L1 ASP transcripts normalized by the RepeatMasker genomic frequencies of L1 subfamilies. Figure S3: YY1 transcription factor binding at L1 ASP transcripts displays two peaks. Binding profiles of YY1 at ESTs supporting L1 ASP transcripts for A) L1-SNCAIP B) L1-KIAA1324L and C) L1-FOCAD. The red indicates the positive strand and the blue indicates the negative strand for the genome browser view. Figure S4: L1 ASP transcripts and accompanying histone modification features in additional cell-lines. A-B) The EST TSS enrichment profile of H3K4me2 and H3K4me3 ChIP-seq data for L1PA2-8 and L1HS subfamilies in H1-ESC and Hela cell lines, respectively. The units for enrichment are input subtracted reads per million mapping reads (RPM). C-D) The EST TSS enrichment profile of GRO-seq data for L1PA2-8 and L1HS subfamilies in MCF7 and Hela cell-lines, respectively. The units for enrichment are reads per million mapping reads (RPM). Figure S5: Examples of L1 ASP transcripts that, despite displaying some evidence of L1 exonization, are likely to be L1 ASP transcripts. A) L1-MAPK10, which primarily displays support for being classified a L1 ASP transcript and minor evidence of L1 exonization. B) L1- SCAMP1, which displays non-overlapping independent support for a L1 ASP transcript and L1 exonization. The red indicates the positive strand and the blue indicates the negative strand for the genome browser view. Figure S6: Genome browser view of conserved L1 ASP transcripts identified in both mouse in human. The gene SHISA5 displays an L1 ASP transcript originated from ancient shared mammalian L1M5 subfamilies. Conservation is displayed for various mammals including Rhesus, Mouse, Rat, Rabbit, Pig, Dolphin, Dog, Elephant, Manatee, and Opossum where dark vertical lines represent conservation. Mammals that contain the L1 ASP in their genome are highlighted in red, whereas blue indicates absence of contributing L1 ASP. (PDF 7039 kb) Additional file 3: Table S2. (excel) Gene ontology analysis for 988 genes that we identified with associated L1 ASP transcripts using Panther online database. (XLSX 11 kb) Additional file 4: Table S3. (excel) Peptide identification for ESTs in Table S1 using Transdecoder. Putative predicted peptides were characterized for the presence of a stop codon and the ORF0 open reading frame. (XLSX 197 kb) Additional file 5: Table S4. (excel) YY1 binding site prediction in L1HS consensus 5’ UTR using online tool JASPAR Scan. (XLSX 11 kb) Additional file 6: Table S5. (excel) Validated Sanger sequences of L1 ASP transcripts L1-KIAA1324L and L1-UVRAG. (XLSX 11 kb) Additional file 7: Table S6. (excel) Overlap between detected putative L1 ASP transcripts from Table S1 and genes identified to play a causal role in cancer (COSMIC database). (XLSX 18 kb) Additional file 8: Table S7. (excel) Summary of ESTs supporting putative L1 ASP transcript where an independent EST supported L1 exonization. These L1 ASP transcripts are considered to be potentially explained by L1 exonization and are of lower confidence. (XLSX 187 kb) Additional file 9: Table S8. (excel) Summary of mouse ESTs (mm10) supporting putative L1 ASP transcripts. Filtering eliminated many putative ESTs because unlike human ESTs much fewer mouse ESTs are labelled with transcript direction information within the intronOrientation field. The 307 ESTs represented in the table sometimes overlap more than one gene and may be represented more than once. (XLSX 36 kb) Additional file 10: Table S9. (excel) Summary of publicly available data used for L1 ASP analysis. (XLSX 14 kb) Additional file 11: Table S10. (excel) Primers used for empirical validation of L1 ASP transcripts. (XLSX 9 kb)
