# Supplementary material for: Genome-wide characterization of human L1 antisense promoter-driven transcripts
Source: BMC Genomics. 2016 Jun 14;17:463. doi: 10.1186/s12864-016-2800-5 (PMC4908685; doi:10.1186/s12864-016-2800-5)

Fig. S1

A) Distribution of ancient L1 ASP transcripts

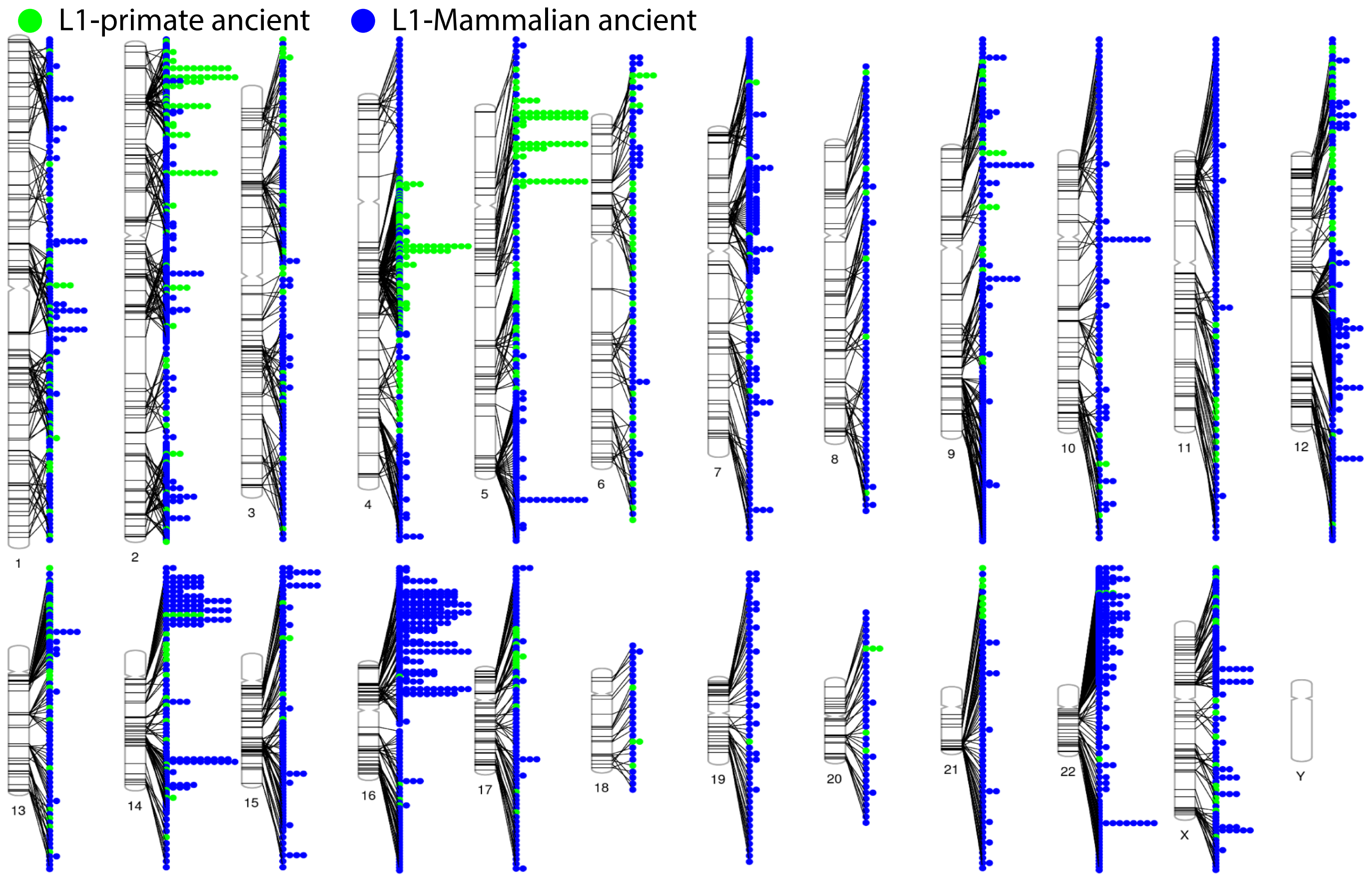

B) Distribution of recent L1 ASP transcripts

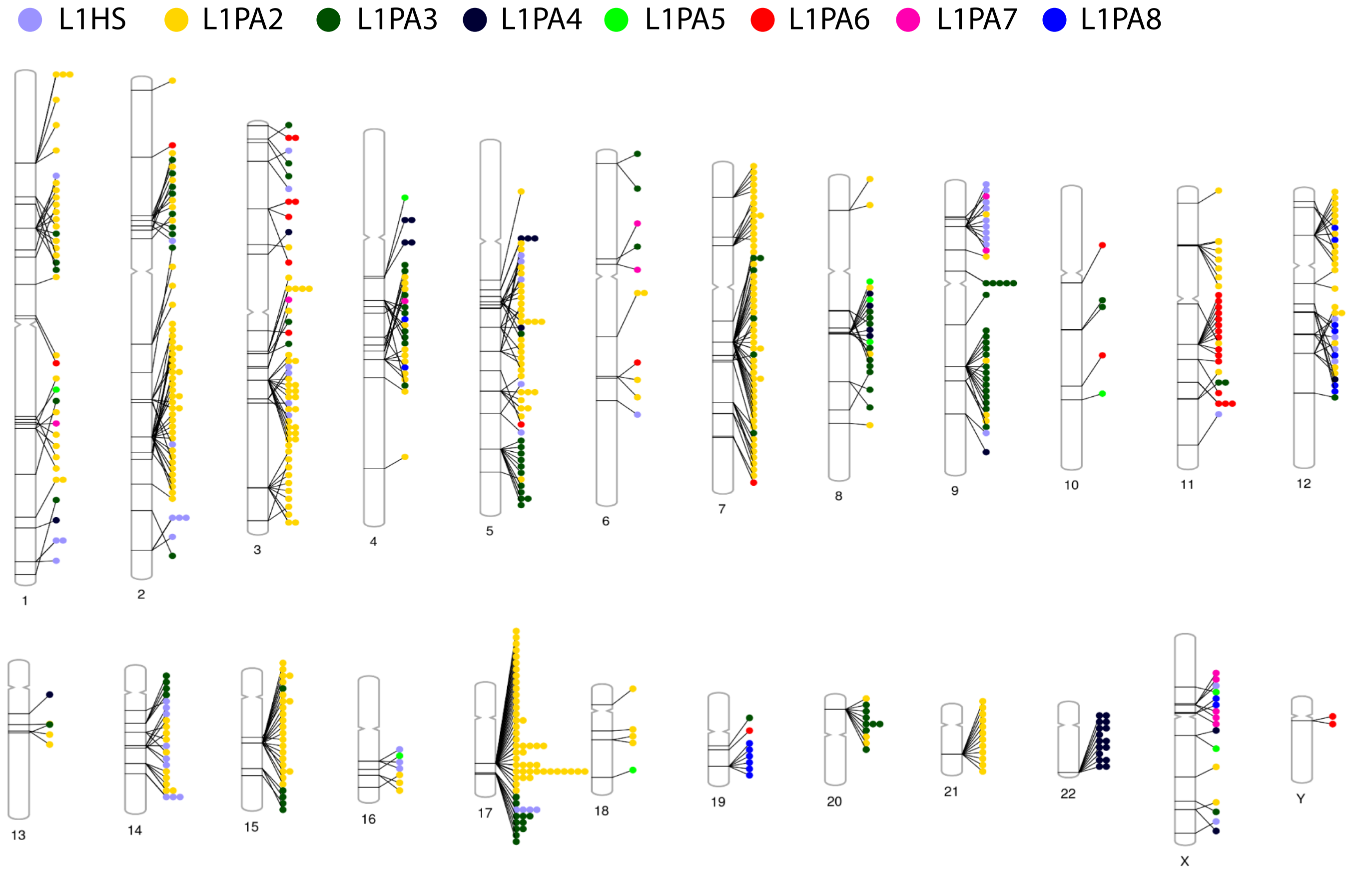

Fig. S2

Normalized L1 subfamily distribution

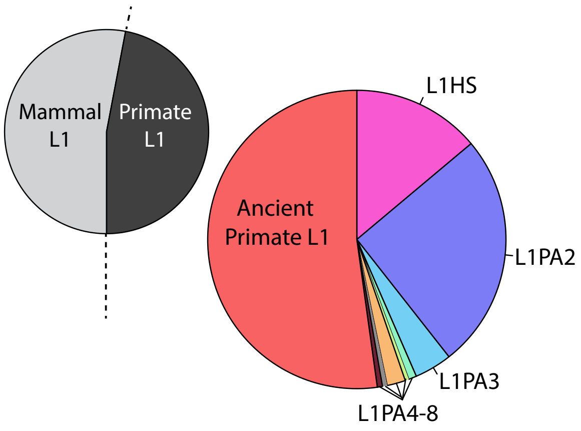

Fig. S3

A) L1-SNCAIP YY1 binding

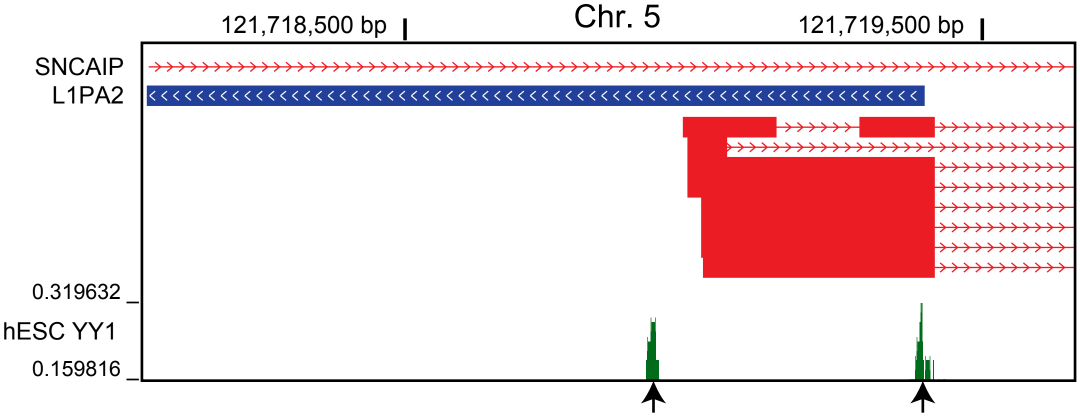

B) L1-KIAA1324L YY1 binding

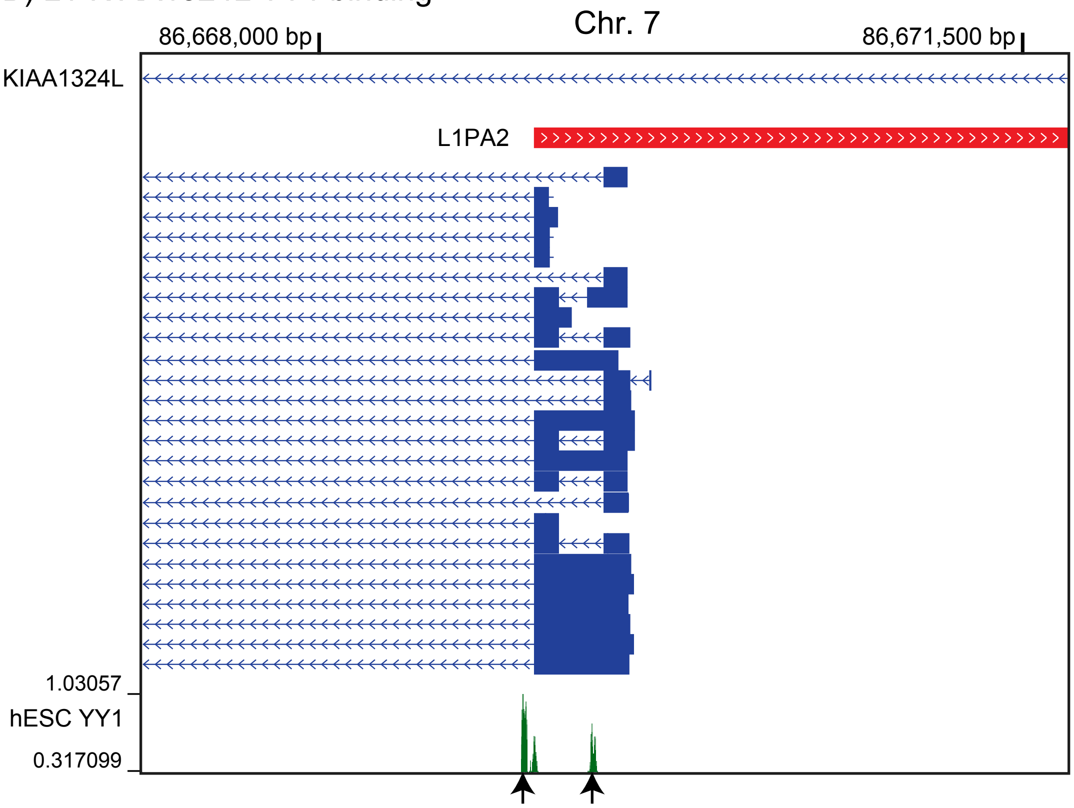

C) L1-FOCAD YY1 binding

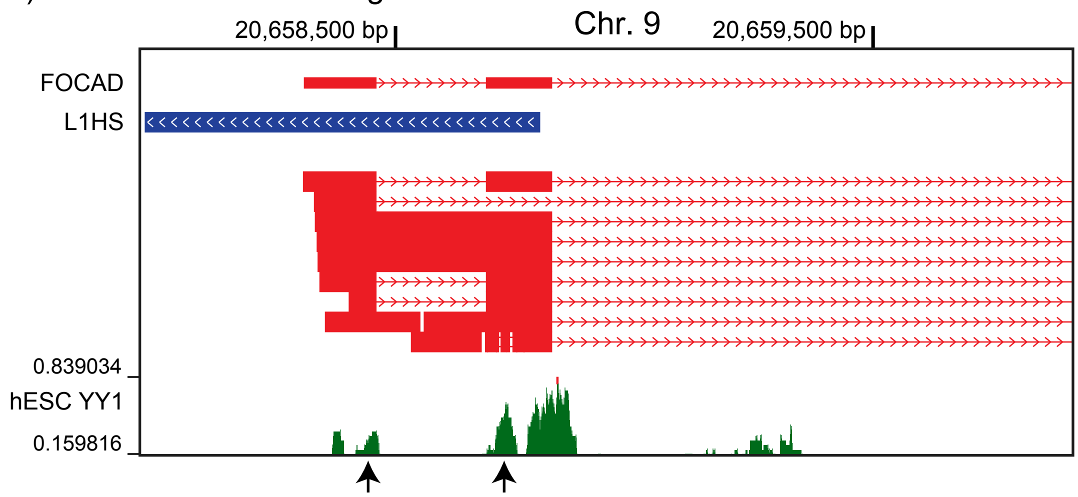

Fig. S4

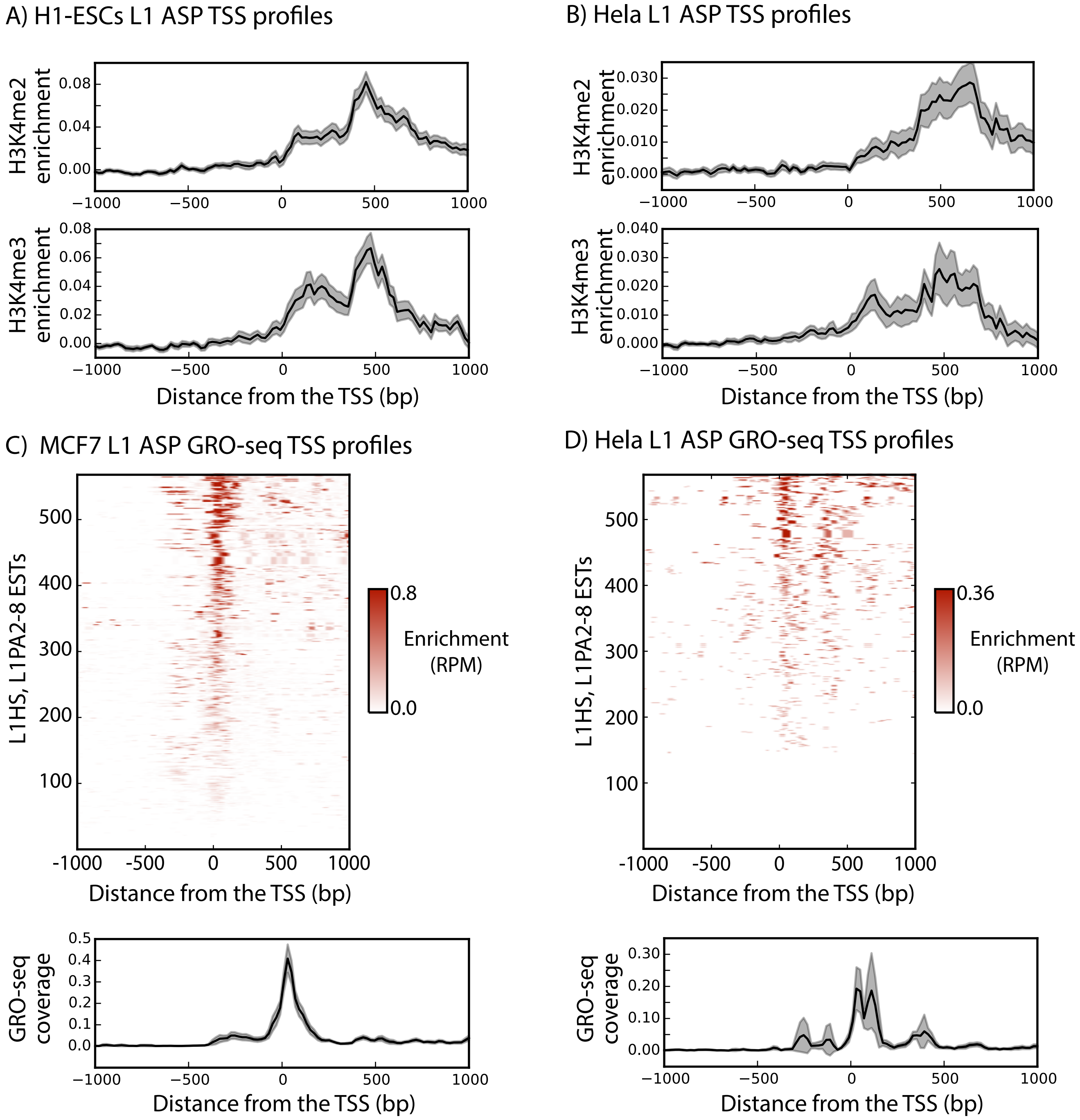
A) H1-ESCs L1 ASP TSS profiles
B) Hela L1 ASP TSS profiles
H3K4me2 enrichment
H3K4me3 enrichment
Distance from the TSS (bp)
C) MCF7 L1 ASP GRO-seq TSS profiles
D) Hela L1 ASP GRO-seq TSS profiles
L1HS, L1PA2-8 ESTs
Enrichment (RPM)
GRO-seq coverage

Fig. S5

A) L1 exonized and ASP transcript MAPK10

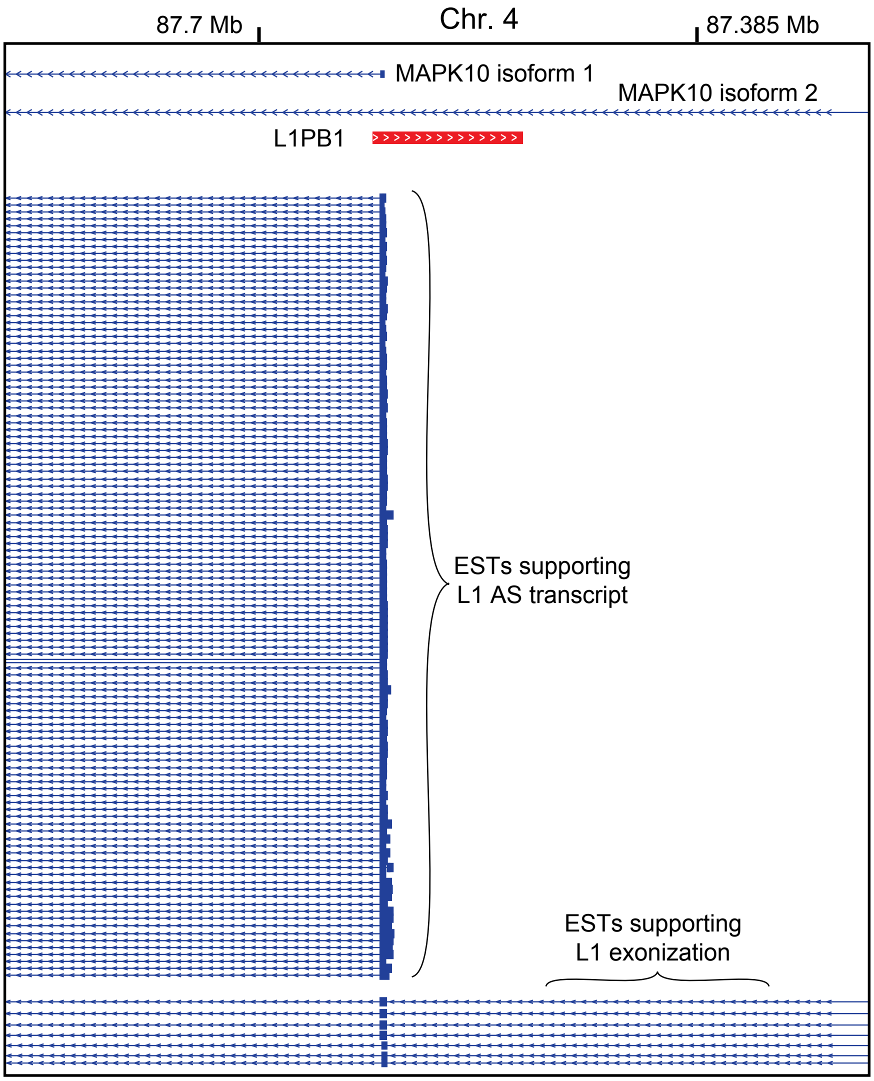

B) L1 exonized and ASP transcript SCAMP1

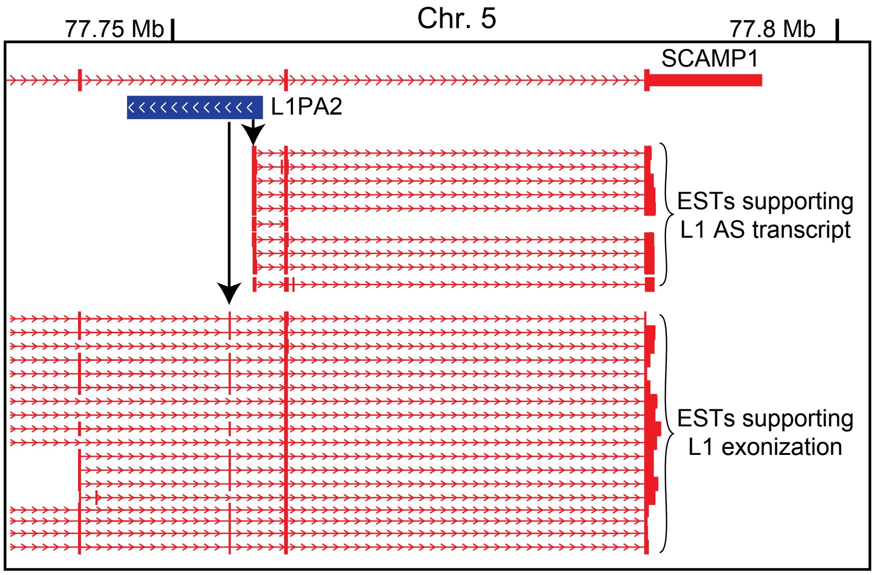

Fig. S6

Conserved L1 ASP transcript

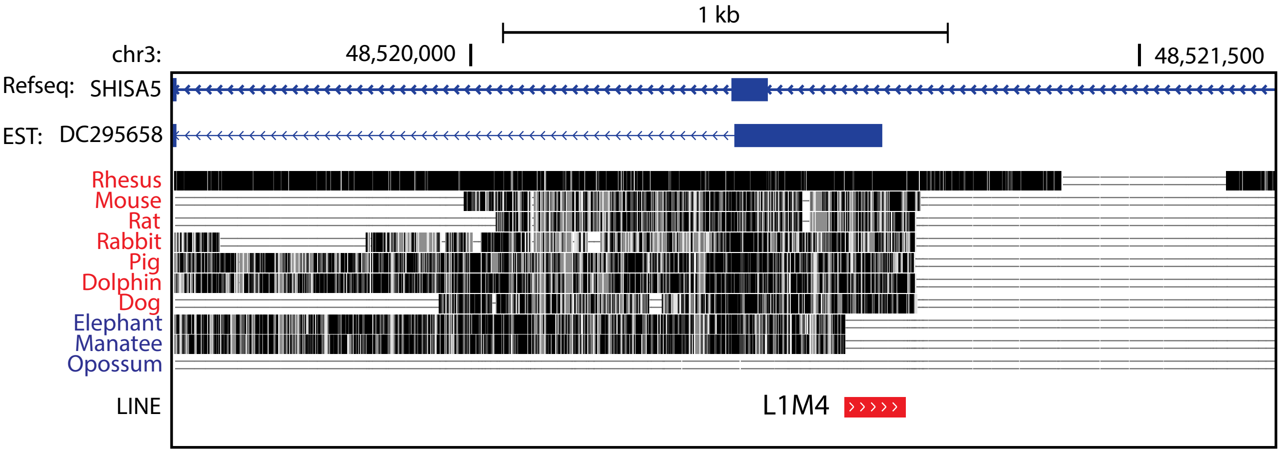

Supplement: Additional file 2: — Supplementary figures 1–6 (PDF). Figure S1. Genomic coordinates of 2015 ESTs supporting L1 ASP transcripts. A) ESTs supporting L1 ASP transcripts from an evolutionarily ancient primate or mammalian L1. B) ESTs supporting L1 ASP transcripts from an evolutionarily recent human-specific L1HS or primate-specific L1PA2-8 subfamily. Figure S2: Evolutionary age of L1 subfamilies that contribute to L1 ASP transcripts normalized by the RepeatMasker genomic frequencies of L1 subfamilies. Figure S3: YY1 transcription factor binding at L1 ASP transcripts displays two peaks. Binding profiles of YY1 at ESTs supporting L1 ASP transcripts for A) L1-SNCAIP B) L1-KIAA1324L and C) L1-FOCAD. The red indicates the positive strand and the blue indicates the negative strand for the genome browser view. Figure S4: L1 ASP transcripts and accompanying histone modification features in additional cell-lines. A-B) The EST TSS enrichment profile of H3K4me2 and H3K4me3 ChIP-seq data for L1PA2-8 and L1HS subfamilies in H1-ESC and Hela cell lines, respectively. The units for enrichment are input subtracted reads per million mapping reads (RPM). C-D) The EST TSS enrichment profile of GRO-seq data for L1PA2-8 and L1HS subfamilies in MCF7 and Hela cell-lines, respectively. The units for enrichment are reads per million mapping reads (RPM). Figure S5: Examples of L1 ASP transcripts that, despite displaying some evidence of L1 exonization, are likely to be L1 ASP transcripts. A) L1-MAPK10, which primarily displays support for being classified a L1 ASP transcript and minor evidence of L1 exonization. B) L1- SCAMP1, which displays non-overlapping independent support for a L1 ASP transcript and L1 exonization. The red indicates the positive strand and the blue indicates the negative strand for the genome browser view. Figure S6: Genome browser view of conserved L1 ASP transcripts identified in both mouse in human. The gene SHISA5 displays an L1 ASP transcript originated from ancient shared mammalian L1M5 s [file 12864_2016_2800_MOESM2_ESM.pdf]
